# Pediatric Pain Management: The Time for Action Is Now

**DOI:** 10.3390/children10121894

**Published:** 2023-12-06

**Authors:** Alessandro Vittori

**Affiliations:** Department of Anesthesia and Critical Care, ARCO Roma, Ospedale Pediatrico Bambino Gesù IRCCS, Piazza S. Onofrio 4, 00165 Rome, Italy; alexvittori82@gmail.com; Tel.: +39-066-859-2397

Pediatric anesthesia is a field of research and assistance in which more specialization needs are emerging [1]. There are now several high-quality studies that underline how the experience of anesthesiologists is one of the factors capable of reducing the incidence of perioperative complications [2,3].

However, the challenge of managing pediatric pain is still present [4,5]—a challenge that can be won but which requires joint efforts from both the scientific community and health policies.

In fact, there are some absolute peculiarities that concern pediatric pain and that require reflection.

First of all, the objective difficulties encountered by researchers dealing with pediatric pain, particularly chronic pain, must be considered [6]. Within the definition of a pediatric patient, there are extremely different patients. Let us think of an adolescent and a newborn. Both of these patients can be defined as pediatric patients, but their needs, and in some cases, their physiology, are not the same [7]. Planning studies is, therefore, difficult and demanding, having to stratify based on age, with cases that necessarily become thinner. All this is even more evident in chronic pain. Chronic pain represents the most difficult challenge for any algologist, especially pediatric ones [8,9]. In chronic pediatric pain, there are all the difficulties of acute pain but with the addition of some peculiar difficulties. Just think of the logistical difficulties. A study on chronic pain involves the need to interview patients or to recall them for follow-up in the pain clinic.

In the case of interviews, the need to obtain information from caregivers can distort the data or make it impossible to collect them. In the case of in-person follow-up, it must be considered that high-level pediatric centers are not as widespread as centers dedicated to adults, so patients often come to that center from remote regions. Recalling a patient for a follow-up aimed only at research is sometimes difficult, if not impossible. Telemedicine could, or rather can, be a tool, together with artificial intelligence, capable of overcoming these barriers and establishing direct, albeit digital, contact with the patient [10].

These research difficulties inevitably translate into objective healthcare difficulties. Most of the drugs available for the treatment of adult pain are off-label in the pediatric field. The pace of production of high-quality literature is unfortunately insufficient [9]. And this is not only true with regards to therapy but also for prevalence studies. It, therefore, seems clear that the pain management of chronic pediatric pain is often a rebus. However, the need for assistance cannot wait: the time for action is now. We need to invest heavily in this field of research and assistance. It is necessary to invest in teams of trained and dedicated health workers. In addition, the role of anesthesiologists within algologists is crucial. In fact, the anesthesiologist is the only algologist who has skills in perioperative medicine, thus being able to cover chronic pain and persistent pain with his co-skills. The figure of the anesthesiologist must be placed at the center of a virtuous system, exactly as happens in intensive care, in which the various pieces of the puzzle are put together by the anesthesiologist. To do this, however, it is necessary to invest in human resources, moving anesthesiologists to a dedicated role in the pain therapy center [11]. Indeed, nurses, psychologists, neurologists, social workers and all the professional figures who, based on their skills, are necessary for multidisciplinary and interdisciplinary treatment of patients are required. It is also necessary to invest in teams of instrumental resources and physical space, since research costs money and requires adequate instruments. If it is true that scientific production in pediatric pain, especially chronic pain, takes a long time, it is also true that it needs to be shortened. If research is essential for care, it is also true that patients’ care needs cannot wait. Chronic pain violently impacts not only the lives of pediatric patients but also their entire families, with significant and sometimes unsustainable social costs, because when a child gets sick, an entire family gets sick. The model of pain as a biopsychosocial phenomenon in the pediatric patient takes on a magnified value, since it actively involves multiple subjects [12].

The WHO establishes the treatment of pain in children as a priority, and not by chance. A lot has been done, but a lot still remains to be done, because patients cannot wait.
